# An Urban Scaling Estimation Method in a Heterogeneity Variance Perspective

**DOI:** 10.3390/e21040337

**Published:** 2019-03-28

**Authors:** Wenjia Wu, Hongrui Zhao, Qifan Tan, Peichao Gao

**Affiliations:** 1 Institute of Geomatics, Department of Civil Engineering, Tsinghua University, Beijing 100084, China; 2 3S Center, Tsinghua University, Beijing 100084, China; 3 National Engineering Laboratory for Green and Safe Construction Technology in Urban Rail Transit, Tsinghua University, Beijing 100084, China; 4 Faculty of Geographical Science, Beijing Normal University, Beijing100084, China

**Keywords:** scaling laws, heterogeneity, China, urban systems, complex systems

## Abstract

Urban scaling laws describe powerful universalities of the scaling relationships between urban attributes and the city size across different countries and times. There are still challenges in precise statistical estimation of the scaling exponent; the properties of variance require further study. In this paper, a statistical regression method based on the maximum likelihood estimation considering the lower bound constraints and the heterogeneous variance of error structure, termed as CHVR, is proposed for urban scaling estimation. In the CHVR method, the heterogeneous properties of variance are explored and modeled in the form of a power-of-the-mean variance model. The maximum likelihood fitting method is supplemented to satisfy the lower bound constraints in empirical data. The CHVR method has been applied to estimating the scaling exponents of six urban attributes covering three scaling regimes in China and compared with two traditional methods. Method evaluations based on three different criteria validate that compared with both classical methods, the CHVR method is more effective and robust. Moreover, a statistical test and long-term variations of the parameter in the variance function demonstrate that the proposed heterogeneous variance function can not only describe the heterogeneity in empirical data adequately but also provide more meaningful urban information. Therefore, the CHVR method shows great potential to provide a valuable tool for effective urban scaling studies across the world and be applied to scaling law estimation in other complex systems in the future.

## 1. Introduction

Urban scaling law, as one of the most significant regulations in urban science [1], has drawn much attention for providing powerful universalities of the variations of urban attributes with city size [2,3,4,5,6]. The urban scaling law state that the relationship between urban attributes and population is well-described by the power-law scaling of the form $y=ax^{b}$ [2], which was inspired by the allometric scaling in biology [7,8]. This law provides a systematic and quantitative framework for urban sustainable development [2,9]. Urban scaling laws reveal empirical regularity for cities across different urban attributes [3,10,11], nations [12,13,14,15], and times [11], validated in both empirical [2] and theoretical [3] ways. Urban scaling studies also provide empirical evidence for the economies of scale and the agglomeration effect beneath the sustainable development of cities [10,16]. Urban scaling laws have been widely applied to analyzing urban hierarchy [17], infrastructure strategies [18], urban designs [19], and environmental strategies [20].

The universality of urban scaling has been questioned in recent studies [21]. It has been observed that the scaling exponent is sensitive to the definition of the city [13], the utilized database [22], and the regression method [23]. For example, the scaling of transportation-related CO2 emissions changes with the population results in different conclusions at different aggregation levels [7]. Opposite conclusions about the foreign investment in the French urban system can be reached using the traditional ordinary least squares (OLS) approach and the computing framework avoiding zero values proposed by Finance [23]. These situations call for more solid statistical support for the study of urban scaling laws.

Challenges still exist for precise statistical estimation methods of the scaling exponent [24,25]. The linear regression method on log-transformed data is utilized widely due to its simplicity, convenience without any iterative process [2,26]. However, the analysis using logarithmic scales involves rotational distortion [27], undetected outliers [28], a multiplicative error without a justification and interpretation [27,29], and a heterogeneous error without justification [30], resulting in a biased estimation result. Researchers suggested that the analysis of urban scaling laws should be carried out on the original scale using a nonlinear regression method [31]. However, heterogeneity of variance has been observed, and the underlying assumption in the regression method is invalid [32]. Furthermore, Leitão [33] argued that challenges should be attributed to statistical properties of cities; namely, the heterogeneous fluctuations of urban attribute Y  as a function of population P. Moreover, the ordinary least squares regression method may result in systematic errors in the power laws [25] and makes an underlying assumption of homogeneous variance without a statistical test. Therefore, the variance of the error model should be considered in urban estimation methods.

The properties of the variance of the error model remain largely unexplored and require further study [26]. Previous studies were interested in obtaining estimates of the urban scaling exponents but did not take enough consideration of the error structure [29]. Both traditional methods utilized the OLS regression with an underlying assumption of homogeneity. However, it has been suggested that heterogeneous variance appeared ubiquitously in complex systems [34,35], and nontrivial fluctuations have been reported in previous studies of urban data [36,37,38]. The heterogeneous variance of the error structure should be considered in the scaling regression, which has been documented in the fields of biology [29] and ecology [32]. It is believed that there exists important information given the heterogeneity of the variance, which should be thoroughly investigated in the models of urban scaling.

In this paper, a statistical framework based on the maximum likelihood estimation considering the lower bound constraints and the heterogeneous variance regression of the error structure, termed as CHVR, is proposed. Traditional approaches for estimating urban scaling parameters are firstly explored, and their limitations are summarized. The properties of heterogeneous variance are explored and integrated into the maximum likelihood-based method. The CHVR method is applied to estimating six urban attributes in China and compared with two traditional methods. Method assessments and a statistical test are performed to validate the CHVR method. Furthermore, the interpretation of the parameter in the variance model is explored to provide extra urban information.

## 2. Study Area and Data 

### 2.1. Study Area

**Figure 1:**
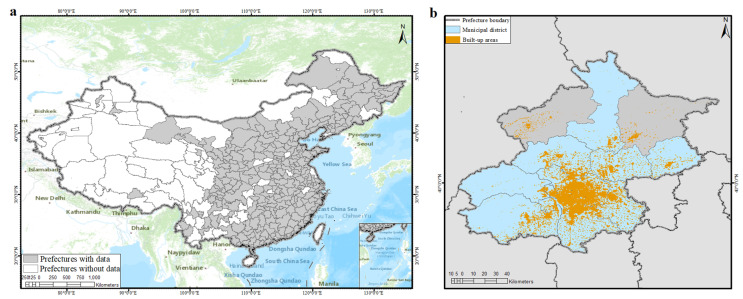
Study areas and statistical units in China in 2010. (**a**) Prefectures in China in 2010. Prefectures in grey represent selected prefectures and prefectures in white, mainly located in the western part, represent prefecture without appropriate data excluded in this study. (**b**) Example of three statistical units related to the definition of the city in the statistical data. Taking Beijing in 2010 as an example, the prefecture in grey refers to the whole Beijing, the municipal district in blue refers to the sum of 16 districts except two counties, and built-up area in orange refers to centralized contiguous parts in the central city and urban construction land with complete infrastructure in suburban areas.

China is considered as the study area for analyzing urban scaling estimation methods, given the rapid urban development [39] and a complex urbanization pattern [40]. Much attention has been given to empirical urban scaling studies in China from different perspectives [41,42]. There are five levels of administrative divisions in mainland China, namely, province, prefecture, county, township, and village. Urban scaling exploration in China needs an appropriate definition of a city, since the urban scaling exponent is sensitive to it, as has been examined theoretically and empirically [13]. There are three statistical units related to the definition of a city in the statistical data, including prefecture (or total city), municipal district (or districts under the city), and built-up area (or built district), as shown in Figure 1a,b. Considering the comparison to Metropolitan Statistical Areas (MSA) in the USA [22], a consistent definition of functional cities, and previous studies in China [15,22,31], urban scaling estimation methods and results are explored using data aggregated at the municipal district (“shixiaqu”) scale for each prefecture. Due to the lack of data and the removing of the inaccurate records, about 275 out of 333 prefectures in mainland China were chosen for the analysis at the municipal district scale, as shown in Figure 1a.

### 2.2. Demographic Data

Demographic data are important for urban scaling as an independent variable. There are two kinds of demographic data in mainland China: household-registered population according to the ‘Hukou’ system, and resident population considering migration. The resident population is more closely related to actual citizens [43] who engage in social interactions and shape city functions; moreover, household-registered population deviates significantly from the resident population for a given city. For example, the size of the household-registered population was only one quarter of the resident population in Shenzhen, China, in 2010. Thus, resident population data at the municipal district level are calculated from the census data in 2010 and are then used to analyze and compare three urban scaling estimation methods in Section 6. Resident population data at the municipal district level in 2000, 2005, 2010, and 2015 are gathered to explore the urban interpretation of the proposed parameter in Section 7.3. Resident population in 2000 and 2010 are provided by censuses, while resident population in 2005 and 2015 are gathered from the China micro-census with the supplement of City Statistical Yearbook and yearly statistical bulletin on national economic and social development for each city at the prefecture level. 

### 2.3. Urban Attributes Data

Statistical information for various urban attributes, namely the Gross Domestic Product (GDP), wage data, water consumption data, electricity consumption data, road area data, and built-up area data at the municipal district level in China for years 2000, 2005, 2010, and 2015 were gathered as the dependent variable in the urban scaling law analysis. These variables were selected with the intention to encompass a range of urban domains in a descriptive way and cover the scaling regimes (super-linear, linear and sub-linear), based on the hypothesis [2,3,10] that super-linear scaling are manifested by quantities related to socioeconomic productivity (GDP, total income), linear scaling are usually associated with basic individual services (household water consumption, household electricity consumption), and sub-linear scaling are manifested by infrastructure demand (built-up area, road surface area). Metadata are collected from the China City Statistical Yearbook. Specifically, GDP at constant price are utilized for comparative studies by year, and paved roads area data are utilized as the measure of road surface area. Then, the necessary pre-processing, including data structuring and deleting outliers, was performed on the metadata to derive the structured urban attributes data. 

## 3. Urban Scaling Laws: Principles and Problems with Estimation

In this section, background knowledge of urban scaling laws is introduced firstly, and then classical approaches to estimating urban scaling parameters and the limitations of such approaches are presented. In addition, heterogeneity of variance of the traditional methods is explored further.

### 3.1. Principles of Urban Scaling Laws

Urban scaling laws [2], also termed as allometric scaling laws for cities, stated that urban attributes Y has a power-law relationship with urban population P, which can be written as:

(1) $$Y=Y_{0}*P^{\beta },$$

where $Y_{0}\;$ is a standardized constant and β is termed as the scaling exponent. Unlike the allometric scaling in biology, whereby all metabolic rates scale sub-linearly with body mass with the exponent of $\frac{3}{4}\;$ [8,44], the scaling exponents β a wide range of urban properties falls into three universal categories [2,3]:

- β>1 shows a super-linear scaling regime associated with social currencies (e.g., GDP, and the number of patents [45]), describing the agglomeration effect. For example, although the population of Beijing is double that of Wuhan, the GDP of Beijing is more than double that of Wuhan.
- β=1 denotes a linear scaling regime associated with individual human needs (e.g., jobs and household water consumption).
- β<1 means a sub-linear scaling regime associated with infrastructural variables (e.g., urban areas and road length), describing economies of scale.

These urban scaling regimes (super-linear, linear, and sub-linear) for urban attributes can reflect non-linear urban development mechanisms, and scaling exponent can provide a measurement of agglomeration effect and scale effect for urban attributes [2,3].

The urban system is regarded as an evolving set of geographically and economically coherent [46] cities at the macroscopic scale [41], characterized by specific behaviors [2]. Empirical studies verified that urban scaling law reflects universal scaling relationships that apply to all urban systems with different levels of development (including the USA [12], Britain [13], Brazil [14], and China [15]). 

In urban scaling estimations, the main question is: given a set of cities, how can the exponent be estimated? Is this scaling super-linear, linear, or sub-linear? Moreover, is the scaling exponent well-evaluated? The scaling exponent has important meanings in urban dynamics studies for the economies of scale and the agglomeration effect beneath the sustainable development of cities. 

### 3.2. Traditional Urban Scaling Statistic Methods

Firstly, the estimation of urban scaling parameters is described in detail from the mathematical perspective. Given a set of cities $\left\{n_{i},\;i=1,2,\ldots ,n\right\}$, the relationship between the set of urban attributes observations $\left\{y_{i},\;i=1,2,\ldots ,n\right\}$ and the set of urban population observations $\left\{p_{i},\;i=1,2,\ldots ,n\right\}$ can be formed as:

(2) $$y_{i}=Y_{0}*p_{i}^{\beta }.$$

However, based on the urban scaling laws, Equation (2) describes the ideal situation where the urban attributes set $\left\{y_{i},\;i=1,2,\ldots ,n\right\}$ and population set $\left\{p_{i},\;i=1,2,\ldots ,n\right\}$ are observed without any error. Considering the reality, we assume that the scaling form in Equation (2) achieves on average. This notion can be formulized by utilizing the conventional mean urban attributes $E\left(y_{i}|p_{i}\right)$, and Equation (2) can be rewritten as:

(3) $$E\left(y_{i}|p_{i}\right)=Y_{0}*p_{i}^{\beta }.$$

There are two common kinds of methods for parameter evaluation in Equation (2). Linear regression based on OLS on logarithmic scales, represented by the LOG_LR method in this paper, is widely used in scaling studies in urban [11] and biology field considering its simplicity [27]. In detail, Equation (2) is log-transformed as linear form as follows:

(4) $$\log\left(y_{i}\right)=\beta *\log\left(p_{i}\right)+\log\left(Y_{0}\right).$$

Then, the linear regression is conducted based on ordinary least squares criterion. That is to say, the error $\varepsilon_{i}$ is assumed to be normally distributed on log-transformed data as:

(5) $$\log\left(y_{i}\right)=\beta *\log\left(p_{i}\right)+\log\left(Y_{0}\right)+\varepsilon_{i},\;\mathrm{and}\;\varepsilon_{i}\sim N\left(0,\sigma^{2}\right),$$

where $\sigma^{2}$ represents the variance of the error model ε. The error $\varepsilon_{i}$ can be referred on the arithmetic scale as follows:

(6) $$y_{i}=Y_{0}*p_{i}^{\beta }{*e}^{\varepsilon_{i}},\;\mathrm{and}\;\varepsilon_{i}\sim N\left(0,\sigma^{2}\right),$$

corresponding to lognormally distributed multiplicative errors. 

The other method applying widely in estimation scaling parameters for urban scaling laws [31] and allometric scaling laws [27] utilized nonlinear regression on the original arithmetic scale, represented by the NLR method. It is assumed that error $\varepsilon_{i}$ is normally distributed as:

(7) $$y_{i}=Y_{0}*p_{i}^{\beta }+\varepsilon_{i},\;\mathrm{and}\;\varepsilon_{i}\sim N\left(0,\sigma^{2}\right).$$

Then, the optimization algorithm, such as the Gauss-Newton method, is conducted to solve the non-linear least squares problems. 

### 3.3. Limitations

As to the LOG_LR method, although linear regression on double-log-transformed data is widely utilized due to its convenience and the lack of recursive iteration, there are four limitations resulting from the transformation to logarithmic scales and a lack of model diagnosis as follows: 

1. The LOG_LR method transforms the power function relating explanatory and response variables into a linear function. Thus, the optimal function has changed in the OLS method, potentially resulting in bias in the estimation. It has been demonstrated that the LOG_LR method predicts geometric means for urban attributes instead of arithmetic means [30]. Log-linear regression based on geometric mean is valuable to the hydrological community [6]; however, scenarios of urban scaling estimations are different from Horton laws originated by entropy maximization [7]. Here, we try to give a statistical justification. From Equation (5), we obtain:

(8) $$E\left(\log\left(y_{i}\right)|p_{i}\right)=\beta *\log\left(p_{i}\right)+\log\left(Y_{0}\right).$$

The conditional probability of $\log\left(y_{i}\right)$ can be written as:

(9) $$P\left(\log\left(y_{i}\right)|p_{i}\right)=\frac{1}{\sqrt{2\pi }*\sigma }*e^{-\frac{{\left(\log\left(y\right)-\mu_{i}\;\right)}^{2}}{2\sigma^{2}}},\;\mathrm{where}\;\mu_{i}=\log\left(Y_{0}\right)+\beta *\log\left(p_{i}\right).$$

Utilizing the integration formula for a compound function, we can derive:

(10) $$P\left(y_{i}|p_{i}\right)=\frac{1}{\sqrt{2\pi }*\sigma }*\frac{1}{y}*e^{-\frac{{\left(\log\left(y\right)-\mu_{i}\right)}^{2}}{2\sigma^{2}}}$$

From Equation (10), it can be inferred that:

(11) $$E\left(y_{i}|p_{i}\right)=\int_{0}^{+\infty }P\left(y|p_{i}\right)*y*dy=\int_{0}^{+\infty }\frac{1}{\sqrt{2\pi }*\sigma }*\frac{1}{y}*e^{-\frac{{\left(\log\left(y\right)-\mu_{i}\right)}^{2}}{2\sigma^{2}}}*y*dy.$$

Omitting the specifics of the integration process, Equation (11) can be computed as:

(12) $$E\left(y_{i}|p_{i}\right)=Y_{0}*p_{i}^{\beta }*e^{\frac{\sigma^{2}}{2}}.$$

Thus, it can be verified that:

(13) $$E\left(y_{i}|p_{i}\right)\neq e^{E\left(\log\left(y_{i}\right)|p_{i}\right)},$$

that is $E\left(y_{i}|p_{i}\right)\neq Y_{0}*p_{i}^{\beta }.$ Therefore, it can be verified that there are transformation biases. 

2. Log-transformation narrows the gap in data; therefore, several outliers may remain undetected [28].

3. Multiplicative errors on the original scale [27] lack statistical justification and meaningful interpretations [29]. 

4. The LOG_LR method makes an underlying assumption that errors are heterogeneous on the arithmetic scale [30], which needs further verification. If the errors are heterogeneous on the arithmetic scale, the weighting may be inaccurate due to a lack of analysis of errors model. Thus, it demonstrated that the estimation of urban scaling parameters should be performed on the original arithmetic scale. 

Regarding the NLR method, however, previous non-linear estimation of scaling laws needs more consideration of the model diagnostics [47] and a common heterogeneous error structure for complex systems.

For both methods, it is only if certain assumptions hold [47] that parameters estimated by the least squares method are consistent with estimators obtained by the maximum likelihood method that has been verified to be more accurate. Therefore, utilizing the least squares method in both traditional methods may introduce some systematic errors [25].

In summary, limitations still exist for both methods, which result from the log transformation, a lack of model structure and statistical justification, and the least squares method. In the following sections, the properties of variance are explored; afterwards, an alternative statistical method based on the maximum likelihood fitting is proposed to estimate the parameters and test urban scaling laws. 

### 3.4. Variance Homogeneity of the Fitting Method

**Figure 2:**
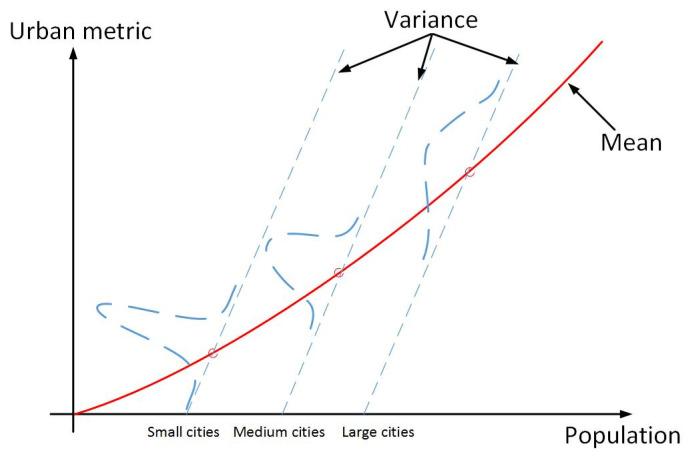
Schematic diagram of heterogeneous variance in the fitting method of urban scaling laws. The red line refers to the fitting method, and the blue dashed lines represent error models with various values of the variance at different values of the independent variable, e.g., for small, medium, and large cities.

Variance homogeneity is one of the important parts of a model structure. Heterogeneity refers to the spread of the data not being the same at each value of the independent variable [32]. The assumption of homogeneity means that conditional on the independent variables, the variance of errors is constant. The alternative means that the variance of errors varies at different values of independent variables, as shown in Figure 2.

**Figure 3:**
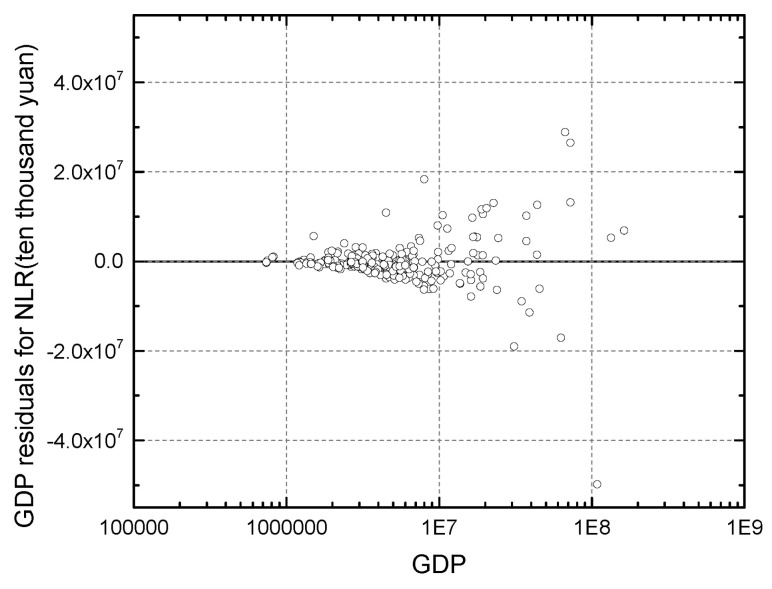
Plot of the log-transformed (to make the effect clearer) fitted values versus the residuals of the Gross Domestic Product (GDP) for the nonlinear regression method.

The homogeneity of variance can be evaluated through a visual examination of residuals. Figure 3 shows the plot of the log-transformed (to make the effect clearer) fitted value versus the residuals for the NLR method. The plot seems to have the shape of a horn with diameter varying from the left to the right. More specifically, as the fitted city attributes increase, it seems that although there is some unevenness in general, it is growing. It can be inferred that the variance is an increasing function of the mean. This finding demonstrates that the assumption of homogeneity of variance is not satisfied. As shown in Figure 2, the variances of adaptive ability differ for cities at different levels of the hierarchical structure of urban systems, including small, medium and large cities. As we know, the population may follow the power law distribution, so that extreme values will occur. There are model violations related to errors being normally distributed with heterogeneous variance [47]. Since a larger population results in larger residuals, the residuals from the larger population will be overestimated compared with those for a relatively small population. Figure 2 and Figure 3 show the tendency of the variation to increase with the mean value [47].

More statistical methods are needed for testing heterogeneity further. If there are available replicated datasets, a statistical test can be performed quantificationally, such as Levene’s test [48]. For urban scaling estimations without replicates, Breusch–Pagan test [49], which tests whether the variance of the residuals is dependent with the independent variables, will be utilized and discussed in Section 6.2.

Therefore, heterogeneous variance is discovered in fitting methods and should be considered in the proposed method for accuracy and to obtain extra urban information [32].

## 4. The CHVR Method

In this section, the maximum probability estimation is utilized to estimate the scaling exponent. The heterogeneous variance will be explored and considered on the error model. The maximum likelihood estimation integrated with the lower bound constraints and the heterogeneity variance regression of error structure, termed as CHVR, is presented in the following sections. 

### 4.1. Maximum-Likelihood Fitting Method Based on Variance Function

For CHVR, the first central question is the likelihood L function of the data. As mentioned in Section 3, in the traditional non-linear fitting method, we assume that the error model is subject to normally distributed, that is:

(14) $$y_{i}=Y_{0}*p_{i}^{\beta }+\varepsilon_{i},\;\mathrm{and}\;\varepsilon_{i}\sim N\left(0,\sigma^{2}\right).$$

Then, the conditional probability P(y|p) has the form:

(15) $$P\left(y|p\right)=\frac{1}{\sqrt{2\pi }*\sigma }*e^{-\frac{{\left(y-\mu \right)}^{2}}{2\sigma^{2}}},\;\mathrm{where}\;\mu =Y_{0}*p^{\beta }.$$

In the CHVR method, heterogeneous variance refers that the variance is dependent on the mean which implies cities with larger population obtain larger errors. Therefore, the heterogeneity will be integrated into the error model, and the variance of the error model is assumed as:

(16) $$\mathrm{var}\left(\varepsilon_{i}\right)=\;\sigma^{2}{\left(Y_{0}*p_{i}{}^{\beta }\right)}^{2\theta },$$

where θ denotes the spread of the variance [50] and typically θ∈[0.5,1] in complex systems [34]. This assumption is motivated by Taylor’s law [51] in the complex science field, and power-of-the-mean variance model [47] in the statistic field. The variance in Equation (16) describes the heterogeneity property, which may be due to mixed effect from the variation of human activity and the fluctuation of data.

Therefore, based on the variance of the error model, we propose the urban scaling law model considering heterogeneity as:

(17) $$y_{i}=Y_{0}*p_{i}^{\beta }+\varepsilon_{i},\;\mathrm{and}\;\varepsilon_{i}\sim N\left(0,\sigma^{2}{\left(Y_{0}*p_{i}{}^{\beta }\right)}^{2\theta }\right).$$

The conditional probability P(y|p) in this method can be computed as:

(18) $$P\left(y|p\right)=\frac{1}{\sqrt{2\pi }*\sigma {\left(Y_{0}*p^{\beta }\right)}^{\theta }}*e^{-\frac{{\left(y-\mu \right)}^{2}}{2\sigma^{2}{\left(Y_{0}*p_{i}{}^{\beta }\right)}^{2\theta }}},$$

where $\mu =Y_{0}*p^{\beta }.$ Based on the definition of the maximum-likelihood fitting method, the likelihood function L can be written as:

(19) $$\begin{array}{cc} L\left(\beta ,Y_{0},\theta ,\sigma |p,y\right) & =P\left(p,y|\beta ,Y_{0},\theta ,\sigma \right)\\ & =P\left(p_{1},y_{1}|\beta ,Y_{0},\theta ,\sigma \right)P\left(p_{2},y_{2}|\beta ,Y_{0},\theta ,\sigma \right)\ldots P\left(p_{n},y_{n}|\beta ,Y_{0},\theta ,\sigma \right), \end{array}$$

that is:

(20) $$L\left(\beta ,Y_{0},\theta ,\sigma |p,y\right)=\prod_{i=1}^{i=n}P\left(p_{i},y_{i}|\beta ,Y_{0},\theta ,\sigma \right).$$

In order to maximize the likelihood function, we log-transformed the Equation (20) as:

(21) $$\log\left(L\left(\beta ,Y_{0},\theta ,\sigma |p,y\right)\right)=\sum_{i=1}^{i=n}\log\left(P\left(p_{i},y_{i}|\beta ,Y_{0},\theta ,\sigma \right)\right).$$

The joint probability density function can be expanded as:

(22) $$P\left(p_{i},y_{i}|\beta ,Y_{0},\theta ,\sigma \right)=P\left(y|p_{i},\beta ,Y_{0},\theta ,\sigma \right)*P\left(p_{i}|\beta ,Y_{0},\theta ,\sigma \right),$$

where $P\left(p_{i}|\beta ,Y_{0},\theta ,\sigma \right)$ denotes the prior probability of the urban population. The prior probability can be obtained by the maximum entropy statistics of the urban system [52,53]. However, it can be suggested that $P\left(p_{i}|\beta ,Y_{0},\theta ,\sigma \right)$ is independent of any of estimators of urban scaling law. Thus, in our method, $P\left(p_{i}|\beta ,Y_{0},\theta ,\sigma \right)$ can be regarded as a constant in the process of fitting, that is:

(23) $$\log\left(L\left(\beta ,Y_{0},\theta ,\sigma |p,y\right)\right)=\sum_{i=1}^{i=n}\log\left(P\left(y_{i}|p_{i},\beta ,Y_{0},\theta ,\sigma \right)\right)+C,$$

where C represents the sum of $\log\left(P\left(p_{i}|\beta ,Y_{0},\theta ,\sigma \right)\right)$, as a constant. Then, Equation (18) can be substituted into Equation (23) and we have:

(24) $$log\left(ℒ\left(\beta ,Y_{0},\theta ,\sigma |p,y\right)\right)=\sum_{i=1}^{i=n}log\left(\frac{1}{\sqrt{2\pi }*\sigma {\left(Y_{0}*p_{i}^{\beta }\right)}^{\theta }}*e^{-\frac{{\left(y_{i}-Y_{0}*p_{i}^{\beta }\right)}^{2}}{2\sigma^{2}{\left(Y_{0}*p_{i}{}^{\beta }\right)}^{2\theta }}}\right)+C.$$

Omitting detailed computing process, Equation (24) can be rewritten as:

(25) $$log\left(ℒ\left(\beta ,Y_{0},\theta ,\sigma |p,y\right)\right)=-n*log\left(\sqrt{2\pi }*\sigma \right)-\sum_{i=1}^{i=n}log\left({\left(Y_{0}*p_{i}^{\beta }\right)}^{\theta }\right)-\frac{1}{2{*\sigma }^{2}}*\sum_{i=1}^{i=n}{\frac{\left(y_{i}-Y_{0}*p_{i}^{\beta }\right)}{{\left(Y_{0}*p_{i}^{\beta }\right)}^{2\theta }}}^{2}+C.$$

Until here, the estimating parameters about urban scaling can be computed by maximizing Equation (25) or minimizing the opposite, which is different from the optimization function in the ordinary least square method. Furthermore, $y_{i}\leq 0$ is included in this method so that observations with $y_{i}\leq 0$ can be considered. However, it has been suggested that observations with $y_{i}<0$ are invalid. Therefore, we are looking forward to a revised method with the lower bound.

### 4.2. Meeting the Lower Bound Constraints

As mentioned previously, for empirical urban data, urban scaling laws are obtained generally only for values of $y_{i}$ above some lower bound $y_{min}$. Therefore, in this section, we build the maximum-likelihood fitting method with the lower bound, where the central question is how to integrate the lower bound into the method.

Firstly, the integral of the conditional probability P(y|p), Equation (18), within the interval $y\in \left[y_{min},+\infty \right]$ can be calculated as:

(26) $$\int_{y_{min}}^{+\infty }P\left(y|p\right)dy=Q\left(\frac{y_{min}-\mu }{\mathrm{var}\left(\varepsilon \right)}\right),$$

where *Q* denotes the *Q* function in statistic, which is the tail distribution function of the standard normal distribution, μ and $\mathrm{var}\left(\varepsilon_{i}\right)$ are generated from Equations (15) and (16), respectively. Since we assume that the probability P(y|p) holds zero for all p<0, the probability P(y|p) for p≥0 in Equation (18) is remained but transformed by multiplying by a standardized function, the reciprocal of Equation (26), to guarantee the entire function is integrated into 1 in the valid domain for urban data. Therefore, we get the conditional probability $\;P\;’\left(y_{i}|p_{i}\right)$ with lower bound as:

(27) $$\;P\;\prime \left(y_{i}|p_{i}\right)=P\left(y_{i}|p_{i}\right)*\frac{1}{Q\left(\frac{y_{min}-\mu_{i}}{\mathrm{var}\left(\varepsilon_{i}\right)}\right)},\;y_{i}>0.$$

Then, the likelihood function $L^{\prime }$ can be described as:

(28) $$L^{\prime }\left(\beta ,Y_{0},\theta ,\sigma |p,y\right)=\prod_{i=1}^{i=n}P{\left(y_{i}|p_{i}\right)}^{\prime }=\prod_{i=1}^{i=n}P\left(p_{i},y_{i}|\beta ,Y_{0},\theta ,\sigma \right)*\frac{1}{Q\left(\frac{y_{min}-\mu_{i}}{\mathrm{var}\left(\varepsilon_{i}\right)}\right)}.$$

In order to maximize the likelihood function, Equation (28) can be log-transformed as:

(29) $$\log\left(L^{\prime }\left(\beta ,Y_{0},\theta ,\sigma |p,y\right)\right)=\sum_{i=1}^{i=n}\log\left(P^{\prime }\left(p_{i},y_{i}|\beta ,Y_{0},\theta ,\sigma \right)\right).$$

Similar to the procedures in Section 4.1, the optimal function in this method can be computed as:

(30) $$log\left(L^{\prime }\left(\beta ,Y_{0},\theta ,\sigma |p,y\right)\right)=-n*log\left(\sqrt{2\pi }*\sigma \right)-\sum_{i=1}^{i=n}log\left({\left(Y_{0}*p_{i}^{\beta }\right)}^{\theta }\right)-\frac{1}{2{*\sigma }^{2}}*\;\sum_{i=1}^{i=n}{\frac{\left(y_{i}-Y_{0}*p_{i}^{\beta }\right)}{{\left(Y_{0}*p_{i}^{\beta }\right)}^{2\theta }}}^{2}-\frac{1}{\sigma^{2}}*\sum_{i=1}^{i=n}Q\left(\frac{y_{min}-Y_{0}*p_{i}^{\beta }}{{\left(Y_{0}*p_{i}^{\beta }\right)}^{2\theta }}\right)+C.$$

However, it can be verified that there is a biased estimation in the method. In this method, the expectation of $y_{i}$ with $p_{i}$ can be computed as:

(31) $$E^{\prime }\left(y_{i}|p_{i}\right)=\int_{y_{min}}^{+\infty }P^{\prime }\left(y|p\right)*y*dy.$$

Omitting detailed computing process, Equation (31) can be rewritten as:

(32) $$E^{\prime }\left(y_{i}|p_{i}\right)=\frac{var\left(\sigma_{i}\right)}{\sqrt{2\pi }}e^{-\frac{\mu^{2}}{2*var{\left(\sigma_{i}\right)}^{2}}}+var\left(\sigma_{i}\right)*Q\left(-\frac{\mu }{var\left(\sigma_{i}\right)}\right)\neq Y_{0}*p^{\beta },$$

where $\;\mu =Y_{0}*p^{\beta }$. Aiming at the unbiased estimation, Equation (32) holds by choosing appropriate parameters $\mu^{\prime }$, which meet the following conditions:

(33) $$E^{\prime }\left(y_{i}|p_{i}\right)=\frac{var\left(\sigma_{i}\right)}{\sqrt{2\pi }}e^{-\frac{{\mu^{\prime }}^{2}}{2*var{\left(\sigma_{i}\right)}^{2}}}+var^{\left(\sigma_{i}\right)}*Q\left(-\frac{\mu^{\prime }}{var^{\left(\sigma_{i}\right)}}\right)=Y_{0}*p^{\beta }.$$

Apparently, there is no analytical solution for both parameters; however, numerical approaches can be achieved cursively in the optimization process.

Then, Equation (33) can be maximized using certain optimization method. In the optimization process, some constraints should be taken into consideration. We can see that the expectation of y with p must be located within the valid interval $\left[y_{min},+\infty \right]$. That is, there is a constraint for the method:

(34) $$E^{\prime }\left(y_{i}|p_{i}\right)=Y_{0}*p_{i}^{\beta }\geq y_{min}.$$

Thus far, the estimating parameters about urban scaling can be computed by maximizing $E^{\prime }\left(y_{i}|p_{i}\right)$ in Equation (33), represented by CHVR. Furthermore, how to choose the lower bound $y_{min}$ will be discussed. Empirically, the urban population has a certain lower bound according to the definition of the city from governments in different countries, for example, the lower limit for the population is 80,000 for China [54], 60,000 for Japan [54], and 50,000 for the USA [55]. To define metropolitan areas, the U.S. Census Bureau also utilizes the population size, density and commuting flow data [56]; however, there is no clear lower limits for urban attributes, such as GDP and wages. Some suggest that $y_{min}$ should be estimated statistically to trade off data discarding against scaling performance, which lacks practical justification and meaning. In our method, $y_{min}$ is set to zero. Moreover, estimators on the condition that $y_{min}$ with other values can also be estimated utilizing our method.

### 4.3. Defining a Self-Starter Function

The initial value is important to the estimating process; thus, a self-starter function is defined to determine the initial value of the parameters in urban scaling laws. Since the log-transformed linear regression method computes the geometric mean of the estimators simply without cursive iterations, the results from the LOG_LR method are utilized as the self-starter function in our method.

## 5. Method Evaluation Criteria

The goodness-of-fit of the fitting method for urban scaling law estimators is usually quantified by the coefficient of determination $R^{2}$, which can be computed as:

(35) $$R^{2}=1-\frac{SS_{res}}{SS_{tot}},$$

where $SS_{res}$ and $SS_{tot}$ denote the sum of squares of residuals and the total sums of squares, respectively. However, the coefficient of determination describes the percentage of the response variable explained by the urban scaling method, which evaluates the statistical significance of the method from one perspective. In addition to $R^{2}$, cross-validation, distance based on Monte Carlo simulation, and the Breusch–Pagan test are also adopted to assess the performance of the proposed CHVR method and provide criteria for compared with two traditional methods, LOG_LR and the NLR method.

For the second evaluation method, cross-validation is performed to evaluate whether the methods can be extended to more generalized datasets [57] avoiding overfitting and selection bias [58]. The specific method of cross-validation is that 80% of the data set was chosen randomly as the training set and the other 20% as the test set; then, the mean squared error (MSE) ${\mathrm{MSE}}_{i}$ is computed to assess the fitting, and this process has been iterated cursively for m times so that the index of the cross-validation for the method, termed as Cross-Validation Index (CVI) can be quantified as:

(36) $$CVI=\frac{1}{m}*\sum_{i=1}^{i=m}MSE_{i}.$$

The cross-validation evaluation method implements the basic assessment out of the limit of independent variables.

The third evaluation method, the Monte Carlo simulation method, is utilized to evaluate the performance of the regression method achieving comparing the simulated and the original data directly [29]. In detail, the target method, such as the CHVR method, is first computed to evaluate the scaling parameters of the empirical dataset. Then, the scaling parameter results can be written as parameter list A. Then n=10,000 synthetic datasets of independent value can be derived from the conditional probability distribution $P^{\prime }\left(y_{i}|p_{i}\right)$ in the method with the fixed parameter sets A via Monte Carlo simulation. Lastly, the average Euclidean distance between the synthetic data sets and empirical data is computed as the goodness-of-fit for the method, termed as the Monte Carlo Index (MCI). The Monte Carlo simulation-based evaluation method implements the basic assessment out of the limit of dependent variables.

The last evaluation method, the Breusch–Pagan test (BP test), is performed to measure heterogeneity of the method. BP test was first put forward by Breusch and Pagan in [49]. In addition to the graphical test for heterogeneity, it can also evaluate if the variance of the residuals of the data is dependent on the variables and provide a valid statistical method. Suppose that we estimate the regression method:

(37) y=f(x,β),

where y represents the dependent variable related to the independent variable through a function f (urban scaling law method), and β denotes the parameter sets. Traditional methods assume that the variance is independent of the variables, termed as the homogeneity assumption. The violation of homogeneity may be referred to as that the variance has a linear relationship with the independent variables, that is:

(38) $$\sigma^{2}=\gamma_{0}+\gamma_{1}x,$$

where $\sigma^{2}$ represents the variance of the disturbance while $\gamma_{0}$ and $\gamma_{1}$ denote the coefficients of this auxiliary regression. Then the null hypothesis of homogeneity can be written as:

(39) $$H_{0}:{\;\gamma }_{0}=\gamma_{1}=0.$$

Two different statistical tests are usually conducted in the BP test [11]. Direct F-test assesses the hypothesis that the error variance does not depend on the independent variables. Indirect Chi-square test is conducted on the auxiliary Lagrange Multiplier (LM) statistic. As the LM test overstates the impact of small or relatively large samples [59], the F-statistic is preferred in our evaluations. 

## 6. Method Evaluations and Results

Method evaluations were performed to compare the performance of the LOG_LR, NLR, and CHVR methods. Three comparing methods are applied to estimating the urban scaling exponents β of six urban attributes in China, including GDP, total income, household water consumption, household electricity consumption, road surface area, and built-up area. The LOG_LR, NLR, and CHVR methods have been compared based on evaluation criteria in Section 5. Furthermore, the standard errors of the estimated β were estimated using bootstrapping with replacement [1]. The summary of the urban scaling exponents, the corresponding standard errors, and method evaluations for these six urban attributes, using the demographic data with high accuracy provided by the census, are demonstrated in Table 1, which were derived from the CHVR method and the traditional methods in China in 2010. 

### 6.1. Method Evaluation Results of the LOG_LR, NLR, and CHVR Methods

The CHVR method presents significant improvements over both LOG_LR and NLR methods in terms of the coefficient of determination $R^{2}$, the Cross-Validation Index (CVI), and the Monte Carlo Index (MCI).

(1) The coefficient of determination $R^{2}$: The value of $R^{2}$ in the CHVR method is the highest, and that in the NLR method is the second highest among these three methods for six urban attributes. Taking the total income as an example, almost 96.25% of the response variable is explained by the CHVR method, compared with 73.67% explained by the LOG_LR method, and 81.45% explained by the NLR method. It can be concluded that the CHVR method shows a significant improvement over traditional methods in terms of $R^{2}$. 

(2) The Cross-Validation Index (CVI): The value of CVI in the CHVR method is much lower than the respective values for the LOG_LR and NLR for urban attributes, clearly indicating that the CHVR method performs better than the other two methods. For urban attributes (namely, GDP, total income, household electricity consumption, and built-up area), it can be observed that CVI in the CHVR is lower than that in the LOG_LR method, and CVI in the NLR method is the highest. For household water consumption, it can be observed that that CVI in the CHVR is lower than that in the NLR method, and CVI in the LOG_LR method is the highest. These comparisons demonstrate that the CHVR method outperforms both LOG_LR and NLR methods in the cross-validation test. Road surface area is a special case, where CVI in the CHVR method is higher than CVI in the NLR method; however, the proposed method still performs better than the LOG_LR method. The selection bias in the dimension of the dependent variable and imprecisions in data gathering may be possible reasons for this exception, which will be further examined. 

(3) The Monte Carlo Index (MCI): The value of MCI of the CHVR method is much lower than the respective values of the LOG_LR and NLR methods for urban attributes, clearly indicating that the CHVR method performs better than the other two methods. For urban attributes, namely, GDP, household electricity consumption, and road surface area, ${\mathrm{MCI}}_{\mathrm{CHVR}}\;<{\mathrm{MCI}}_{\mathrm{NLR}}<{\mathrm{MCI}}_{\mathrm{LOG}\_\mathrm{LR}},$ demonstrating that the CHVR method performs the best, and the NLR method performs second best among the three methods. For urban attributes, namely, total income, household water consumption, and built-up area, it can be observed that ${\mathrm{MCI}}_{\mathrm{CHVR}}\;<{\mathrm{MCI}}_{\mathrm{LOG}\_\mathrm{LR}}<{\mathrm{MCI}}_{\mathrm{NLR}}$, showing that the CHVR method performs the best, and the LOG_LR method performs second best among the three methods. It can be concluded that the CHVR method can derive urban scaling exponents with higher accuracy in terms of MCI out of limits of dependent variables. 

In summary, the results obtained using three kinds of comparison criteria verify the outperformance of the CHVR method in terms of a basic statistical assessment, an assessment based on random independent variables, and dependent variables. The effectiveness of the CHVR method is mainly attributed to the variance function with heterogeneity describing mixed effects accurately and the consideration of the lower bound constraints.

### 6.2. Heterogeneity Results of the LOG_LR, NLR, and CHVR Methods

To further explore the effectiveness of the variance function describing heterogeneity in the CHVR method, a visual inspection of residuals and the BP test were utilized to examine how heterogeneity varies between the traditional methods and the CHVR method.

**Figure 4:**
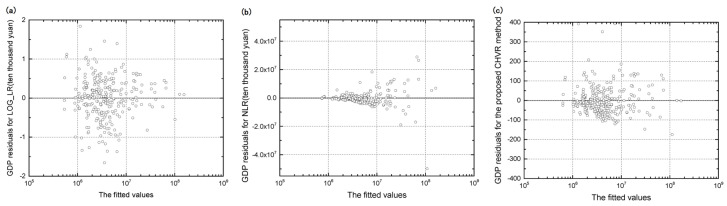
Plot of the log-transformed fitted values versus residuals of the GDP for (**a**) the LOG_LR method, (**b**) the NLR method, and (**c**) the CHVR method.

The plot of the log-transformed fitted values versus residuals of urban attributes was utilized to compare heterogeneity in three methods qualitatively. Taking GDP as an example, as shown in Figure 4, the plots of the log-transformed fitted values versus residuals of the GDP for both LOG_LR and CHVR methods show that the residuals are independent of the fitted value, indicating homogeneity. However, the shape of a horn for the NLR method indicates heterogeneity, as described in Section 3.4.

Furthermore, the BP test was implemented to test the heterogeneity between the LOG_LR, NLR, and CHVR methods quantificationally. The F-statistics values and their corresponding *p*-values are listed in Table 2. Sequential Bonferroni [60] was utilized to control the experiment-wise Type I error rate. The Sequential Bonferroni method sorts the *p*-values in ascending order and compares them to nominal alpha levels of $\frac{\alpha }{m}$ to α, where α denotes the desired overall alpha level and m is the number of hypotheses. In detail, as for GDP, if α=0.05  is assumed as the overall alpha level, the *p*-values for the F-statistics in three methods are firstly sorted from small to large, and then, homogeneity hypothesis is tested at a significance level of $\frac{0.05}{3}$ for the method with the smallest *p*-value, $\frac{0.05}{2}$ for the method with medium *p*-value, 0.05 for the biggest. If the *p*-value for the F-statistics is greater than the testing significance level, the null hypothesis can be accepted so that homogeneity appeared in this method according to the BP test. Otherwise, heterogeneity can be concluded and marked with an asterisk ‘*’.

It is noteworthy that the homogeneity assumption was accepted for the CHVR method for all six urban attributes. In contrast, the homogeneity assumption was rejected for the NLR method for all six urban attributes; in addition, as for the LOG_LR method, the homogeneity assumption was accepted for four urban attributes (GDP, total income, road surface area, and built-up area), but not for household water and electricity consumption. Specifically, significant heterogeneity is observed in the NLR method, which reduces the accuracy of exponent estimation. In the LOG_LR method, an underlying heterogeneity assumption in the linear regression method on the logarithmic scale can account for homogeneous results for four urban attributes. Considering that heterogeneity is not described by a specific variance model, but rather by the underlying result of log-transformation, heterogeneity is not characterized effectively for household consumption attributes; thus, the homogeneity assumption is rejected. Furthermore, it is assumed that the larger the *p*-value is for the F-statistics that corresponds to the null hypothesis of homogeneity, the more homogeneous the residuals of the model. The highest *p*-value in the CHVR method for five urban attributes other than the total income shows that significant heterogeneity has been eliminated through the variance function in the CHVR method. As to the total income, a lower *p*-value in the CHVR method compared with that of the LOG_LR method may be ascribed to the inappropriate description of heterogeneity, while the NLR method is more appropriate. In essence, the heterogeneity can be appropriately described in our framework.

The above suggests that the variance function in the CHVR method can describe heterogeneity adequately and, compared with traditional estimation methods, results in a more efficient estimation of urban scaling exponents.

## 7. Discussions

In this section, scaling functions for six urban attributes based on the CHVR method are analyzed to explore the urban development mechanisms in China; then, exponents among three methods are discussed regarding how the estimated exponent depends on the method. In addition, interpretation of the parameter in the variance model is explored from a dynamic perspective to provide extra urban information.

### 7.1. Urban Scaling Law in CHINA Based on the CHVR Method

The similarities and differences of urban scaling regimes for six attributes in China compared with developed countries quantificationally revealed the urban development trends in China.

The exponents of urban scaling for some of the urban attributes in China fall into the same scaling regimes as those of other countries in the previous studies [3,61], including super-linear scaling for socioeconomic attributes, linear scaling for household-related attributes and sub-linear scaling for infrastructure-related attributes. However, there are also some unique phenomena of exponents of the urban scaling laws in China as shown in Table 1. As to socioeconomic attributes, the GDP scaling exponent of China is 1.2022, representing super-linear scaling and indicating the urban agglomeration effect in the economy in China. Furthermore, the exponent is greater than the expected value of $\frac{7}{6}$ proposed by Bettencourt [3] and 1.11 in the United States [3]. This finding indicates that urban population agglomeration contributed more to China’s economic growth in 2010. A similar phenomenon has also been explored for the total income with $\beta_{CHVR}=1.2032$. Considering household-related attributes, both the household water consumption and the electricity consumption fall into the super-linear regime instead of the expected linear regime, with exponents of 1.1131 and 1.1484, respectively. This finding may be attributed to lower energy consumption efficiency characterizing the stage of extensive economic growth in China in 2010. As to infrastructure-related attributes, the built-up area’s scaling exponent in China is 0.8790 and greater than the theoretically expected value of 5/6 and 0.6~0.8 in developed countries [3]. The comparative exponents results indicate that the built-up areas in China display weaker scale effects than developed countries and the optimized situation proposed by Bettencourt [3]. Thus, this finding indicates lower built-up land use efficiency compared with developed countries and an unbalanced relationship between land urbanization and population urbanization in China in 2010. The policies for intensive land use and optimization of land use structure could be a better solution in the process of urbanization in China, consistent with “economical and intensive land use” policies in China proposed in 2008 and developed in 2012 [15,16]. The scaling exponent of the road surface area is 0.9471, far greater than the theoretical value of 5/6 [3] and even closer to 1. This exponent indicates a nearly linear scaling relationship between road surface area and population size. Some may argue that this relationship is ambiguous between linear and sub-linear scaling. However, it can be observed that this ambiguous scaling relationship does not reflect the economies of scale for the road surface area in previous studies in developed countries [19]. The observed exponent suggests that the total scale of road infrastructure construction has become large enough, but the efficiency is not optimal, and there is a lack of cost advantages of the economies of scale. This may result from the unbalanced relationship between road infrastructure supply (e.g., road network and transport infrastructure structure) and demand (e.g., urban morphology and land use pattern).

### 7.2. Dependence of the Estimated Exponent on the Method

How the scaling exponent depends on the estimation method is discussed from both the urban attributes dimension and the estimation method dimension. 

**Figure 5:**
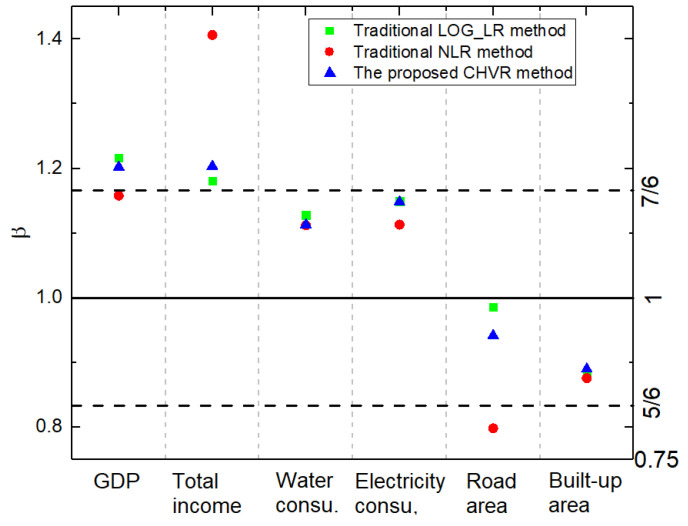
Comparison of results for urban scaling exponents for the LOG_LR, NLR, and CHVR method in China in 2010. The green squares, red circles, and blue triangles denote the scaling exponents calculated from the LOG_LR, NLR, and CHVR methods, respectively. The dotted lines refer to the theoretically expected value [3] $\beta =\frac{7}{6}$ for social currency-related attributes (the super-linear regime), β=1 for human needs-related attributes (the linear regime), and $\beta =\frac{5}{6}$ for infrastructure-related attributes (the sub-linear regime), respectively.

**Figure 6:**
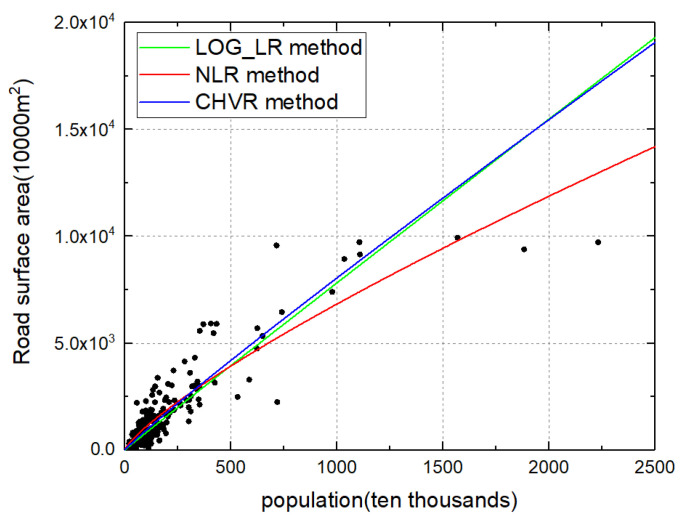
Different urban scaling laws of road surface area from the LOG_LR, NLR, and CHVR methods.

From the urban attributes dimension, the dependence of the exponent on the estimation method exists in different urban attributes. There are different magnitudes of variations observed for different urban attributes under study. Exponents of household water consumption and the built-up area show less dependence on the estimation methods, while exponents of other urban attributes (GDP, total income, household electricity consumption, road surface area) fluctuate widely for different estimation methods, as shown in Figure 5. For instance, the scaling exponent of the total income in the NLR method is much larger than the exponent in the CHVR and LOG_LR method. Therefore, different estimation methods will result in different scaling regimes for these urban attributes. Considering the road surface area, the scaling exponents were less than 1 in the CHVR and NLR method, indicating a sub-linear scaling regime for this urban attribute and corroborating the expected scaling regime [3]. However, the scaling exponents obtained from the LOG_LR were approximately 1, indicating a nearly linear instead of sub-linear scaling relationship. The different urban scaling laws of the road surface area in the three methods are illustrated in Figure 6. Furthermore, different estimation methods will result in different numerical relationships with theoretically expected values of these urban attributes. In particular, for the total income, the estimated exponents in the LOG_LR method and the CHVR method were close to the theoretical value $\beta =\frac{7}{6}$; however, the exponent in the NLR method was far above the expected value. The dependences on the estimation method for different attributes have important theoretical and empirical implication for urban science. The universality of urban scaling law has been doubted due to inconsistent results obtained for different countries [13,21] compared with theoretical and empirical regimes reported by Bettencourt [2,3]. However, this discrepancy may occur affected by the urban scaling estimation method. For example, the scaling exponent of the road surface area obtained using the CHVR method complies with urban scaling law, even though there is a paradox for the LOG_LR method. In addition, the urban scaling exponent β is affected by not only the estimation method but also the urban definition, i.e., the data aggregation scale. Therefore, urban scaling regimes cannot be obtained independently from the analysis of the variance of the error model in the estimation method.

From the estimation method dimension, the exponent from the CHVR method locates in the middle of the LOG_LR method and NLR method, indicating that the variance of error model in the proposed method is analogous to a weighted model of the variance in LOG_LR and NLR methods. It can be observed that there is certain comparative relationship among three methods, i.e., the exponent obtained using the CHVR method is larger than that of one traditional method and smaller than that of the other. As shown in Figure 5, blue dots are located in the middle of red and green dots for all urban attributes apart from the built-up area, where the exponents from three methods were nearly consistent. The LOG_LR method assumes the lognormally distributed and multiplicative error by default, and the NLR method assumes the normally distributed and additive error by default. In our method, properties of variance have been explored and derived as the power-of-the-mean variance model. This result indicates that our method is analogous to a weighted model of LOG_LR and NLR methods. In other words, the CHVR method is not an ordinary weighted model of two traditional models. This model can be viewed as an intermediate state between the LOG_LR and NLR models. It is analogous to that of a small-world network being an intermediate state between the rule network and the random network [62] in the complex network field. Similarly to the small-world network, the CHVR method is based on a heterogeneous model of variance in real-world urban systems, where the error is not purely multiplicative or additive, and variances for different residuals are not purely dependent or independent. Thus, our method can effectively describe not only the combination of different effects, such as the variation of human activity and the fluctuation of data, but also the intermediate state originating from the real-world urban complex system, such as the error model and the relationship between variances for different residuals. 

### 7.3. Urban Interpretations of θ from a Dynamic Perspective

**Figure 7:**
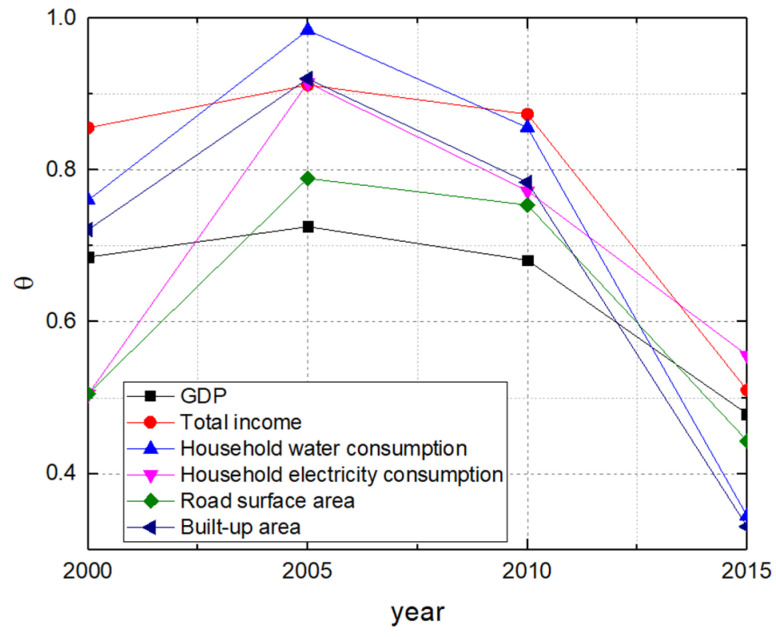
The values of θ are derived by the CHVR method for all six urban attributes for the year 2000, 2005, 2010, and 2015.

In this section, the interpretation of the parameter θ in the variance function of the CHVR method is explored to discover extra urban information beneath the heterogeneity in urban scaling law. The parameter θ was calculated in the long term for all six urban attributes. Specifically, as shown in Figure 7, the values of θ were derived by the CHVR method for all six urban attributes for the year 2000, 2005, 2010, and 2015 considering the urban development process in China and data limits described in Section 3. The variation of the parameter θ is examined to understand the meaning of this parameter in urban systems.

Regarding all six attributes belonging to three scaling regimes, the value of the parameter θ in 2005 was higher than that in 2000, and then it decreased continuously from 2005 to 2015, as shown in Figure 7. For instance, for GDP, the value of parameter θ increased from θ=0.6855 in 2000 to θ=0.7255 in 2005, when the value reached the maximum; then, the value of parameter θ declined to $\theta_{2010}=0.6813$ in 2010 and $\theta_{2015}=0.4788$ in 2015. The peak in 2005 shows that there was a transition point around 2005 in the process of urban development in China so that the urbanization in China can be categorized into two stages according to the value of θ. This result is consistent with the findings by Chen [63], who discovered that urbanization velocity attained a peak in the year of 2004 or 2005 bringing two stages in the process of China’s urbanization, as well as the statistical results from China Statistical Yearbook, where the peak arrives at around 2003. Statistically, the high value of the parameter θ means overdispersion within the data. It was suggested by West and Bettencourt [64] that the greater degree of dispersion in urban systems, which is in the self-organized evolution process, represents the shorter time for the urban systems to move toward the ideal optimal configuration represented by the scaling law. Therefore, the continuous reduction of θ since 2005 shows less dispersion over time after 2005, and further implies that urban system in China moves toward the ideal optimal configuration represented by the scaling law unceasingly from 2005 to 2015. In addition, the unexpected growth of θ from 2000 to 2005 may be attributed to that self-organized behaviors appear, so that an urban scaling effect is not apparent.

The variation of the parameter θ indicates that the exponent θ in urban systems can be interpreted as not only the degree of overdispersion statistically but also the index of matureness of the self-organized urban systems toward the ideal configuration represented by the scaling law, which is related with urbanization velocity, inequality between large cities and small cities, urban efficiency, and spatial synchronization. The interpretation of the variance function in the form of Taylor’s Law has remained of interest over time, with particular effort directed to understanding the exponent θ in different complex systems [37]. The meaning of θ has been interpreted to different meanings depending on the system studied, synchronization in transportation system [65], randomness and aggregation in ecology systems [51], and population growth in human geography systems [36]. 

## 8. Conclusions

Urban scaling law is the key to establishing a predictive and quantitative theory for urban organization and sustainable development. Although urban scaling law has been explored theoretically and empirically in previous work, accurate scaling exponent estimation is still challenging. Limitations exist in the traditional methods, and the variance structure need be explored further.

In this paper, a maximum likelihood-based statistical framework named CHVR is proposed to estimate the urban scaling exponents considering both the lower bound constraints and the error structure of heterogeneity. The CHVR method has been applied to estimating the scaling exponents for six urban attributes in China. Method evaluations have been performed utilizing the coefficient of determination, the cross-validation index, and Monte Carlo-based index. Evaluation results show that all three evaluation indexes for the CHVR method yield improvement over the LOG_LR and NLR methods for all six urban attributes (e.g., GDP, total income, household water consumption, household electricity consumption, road surface area, and built-up area). The properties of variance heterogeneity were explored through the Breusch–Pagan test, demonstrating that the variance function in the CHVR method describes heterogeneity adequately and results in a more effective estimation of urban scaling exponents than do traditional estimation methods. The variation of parameter θ in the long term shows that exponent θ implies not only the overdispersion degree in statistics but also an index of maturity for self-organized urban systems to the optimal configuration represented by the scaling law. It can be concluded that the CHVR method is more effective and robust than traditional scaling estimation methods and has great potential in analyzing urban development mechanisms.

Future research is recommended in the following areas. Firstly, other possible forms of methods considering heterogeneous variance can be explored. Secondly, the CHVR method can be applied to more countries, urban attributes, and periods of time. Thirdly, the variation of θ in finer granularity time can be explored to further unearth the interpretation of θ. Finally, this study has been conducted at the municipal district scale in China, and empirical studies can be applied to more spatial scales.

## Figures and Tables

**Table 1:** Summary of the urban scaling exponents and method evaluation to three methods.

Urban Attributes	Methods	β	*SE*	θ	$R^{2}$	CVI	MCI
GDP	LOG_LR	1.2162	0.0345	/	0.7814	$3.4616\;\times \;10^{13}$	$2.1376\;\times \;10^{8}$
	NLR	1.1580	0.0591	/	0.9099	$3.5702\;\times \;10^{13}$	$2.0037\;\times \;10^{8}$
	CHVR	1.2022	0.0300	0.6813	**0.9342**	**3.3173 × 10^13^**	**1.2806 × 10^8^**
Total income	LOG_LR	1.1809	0.0397	/	0.7367	$3.1409\;\times \;10^{12}$	$3.3998\;\times \;10^{7}$
	NLR	1.4063	0.2401	/	0.8145	$3.6023\;\times \;10^{12}$	$4.7743\;\times \;10^{7}$
	CHVR	1.2032	0.0395	0.8737	**0.9625**	**2.4320 × 10^12^**	**3.0365 × 10^7^**
Household water consumption	LOG_LR	1.1278	0.0377	/	0.7283	$2.2582\;\times \;10^{7}$	$1.2999\;\times \;10^{5}$
	NLR	1.1121	0.0727	/	0.8389	$2.2155\;\times \;10^{7}$	$1.5787\;\times \;10^{5}$
	CHVR	1.1131	0.0332	0.8561	**0.9346**	**2.0520 × 10^7^**	**1.0287 × 10^5^**
Household electricity consumption	LOG_LR	1.1493	0.0331	/	0.8278	$2.4318\;\times \;10^{9}$	$1.6676\;\times \;10^{6}$
	NLR	1.1130	0.0518	/	0.9371	$2.6383\;\times \;10^{9}$	$1.6592\;\times \;10^{6}$
	CHVR	1.1484	0.0310	0.7539	**0.9574**	**2.2693 × 10^9^**	**1.0758 × 10^6^**
Road surface area	LOG_LR	0.9862	0.0287	/	0.7034	$8.9181\;\times \;10^{5}$	$3.0466\;\times \;10^{4}$
	NLR	0.7984	0.0430	/	0.8155	**6.7414 × 10^5^**	$2.8398\;\times \;10^{4}$
	CHVR	0.9417	0.0236	0.7727	**0.8573**	8.8028 × 10^5^	**2.0146 × 10^4^**
Built-up area	LOG_LR	0.8790	0.0287	/	0.7544	$3.2347\;\times \;10^{3}$	$1.5083\;\times \;10^{3}$
	NLR	0.8758	0.0544	/	0.8790	$3.4481\;\times \;10^{3}$	$1.9226\;\times \;10^{3}$
	CHVR	0.8900	0.0273	0.7840	**0.8882**	**2.9697 × 10^3^**	**1.2085 × 10^3^**

Note: SE denotes the standard errors of the estimated β. Each evaluation index marked with bold indicates the best performance among three methods for the urban attribute according to the evaluation criterion.

**Table 2:** Summary of F-statistics results in the BP test for the LOG_LR, NLR, and CHVR method for six urban attributes.

Parameter	LOG_LR			NLR			**CHVR**	
	F-Value	*p*-Value		F-Value	*p*-Value		**F-Value**	***p*** **-Value**
GDP	3.3241	0.0694		48.9939	1.99 × $10^{-11}$ *		0.6134	0.4341
Total income	0.0237	0.8778		35.2855	8.64 × $10^{-9}\;$*		3.3044	0.0702
Water consumption	6.5394	0.0111*		40.9598	6.74 × $10^{-10}\;$*		0.7598	0.3842
Electricity consumption	5.6067	0.0186*		46.8604	5.15 × $10^{-11\;}$*		0.0579	0.8101
Road surface area	2.2672	0.1333		91.3284	7.44 × $10^{-19}\;$*		0.2567	0.6128
Built-up area	0.3846	0.5357		81.3017	3.51 × $10^{-17}\;$*		0.1677	0.6825

Note: Water consumption and electricity consumption denote household water consumption, and household electricity consumption, respectively. Those *p*-values less than the testing significance level are marked with an asterisk ‘*’ indicating heterogeneity.
